# MicroRNA and Human Bone Health

**DOI:** 10.1002/jbm4.10115

**Published:** 2018-11-05

**Authors:** Vincent Ka‐Fai Cheng, Philip Chun‐Ming Au, Kathryn CB Tan, Ching‐Lung Cheung

**Affiliations:** ^1^ Department of Pharmacology and Pharmacy The University of Hong Kong Pokfulam Hong Kong; ^2^ Department of Medicine The University of Hong Kong Pokfulam Hong Kong; ^3^ Centre for Genomic Sciences Li Ka Shing Faculty of Medicine The University of Hong Kong Pokfulam Hong Kong

**Keywords:** MICRORNA, OSTEOPOROSIS, METABOLISM, PolymiRTS

## Abstract

The small non‐coding microRNAs (miRNAs) are post‐transcription regulators that modulate diverse cellular process in bone cells. Because optimal miRNA targeting is essential for their function, single‐nucleotide polymorphisms (SNPs) within or proximal to the loci of miRNA (miR‐SNPs) or mRNA (PolymiRTS) could potentially disrupt the miRNA‐mRNA interaction, leading to changes in bone metabolism and osteoporosis. Recent human studies of skeletal traits using miRNA profiling, genomewide association studies, and functional studies started to decipher the complex miRNA regulatory network. These studies have indicated that miRNAs may be a promising bone marker. This review focuses on human miRNA studies on bone traits and discusses how genetic variants affect bone metabolic pathways. Major ex vivo investigations using human samples supported with animal and in vitro models have shed light on the mechanistic role of miRNAs. Furthermore, studying the miRNAs’ signatures in secondary osteoporosis and osteoporotic medications such as teriparatide (TPTD) and denosumab (DMab) have provided valuable insight into clinical management of the disease. © 2018 The Authors. *JBMR Plus* Published by Wiley Periodicals, Inc. on behalf of the American Society for Bone and Mineral Research

## Introduction

Osteoporosis is characterized by reduced bone strength and deteriorated bone microarchitecture. One‐third of women and one‐fifth of men worldwide, aged 50 years and older, are expected to experience osteoporotic fracture.^1^, ^2^ The subsequent health care cost regarding hospitalization and related follow‐up treatment is surging every year. In Europe, the health care cost resulting from osteoporosis is expected to increase by 10 billion euro over a 15‐year period from 2010 to 2025.^2^ This would pose an immense financial burden to society, thus making osteoporosis treatment and prevention an important research area.

Currently, the usual approaches for screening and monitoring approaches for osteoporosis include the dual‐energy X‐ray absorptiometry (DXA) and FRAX score. Although DXA is the gold standard for osteoporosis diagnosis, predicting fracture based solely on bone mineral density (BMD) value has been unreliable.^3^, ^4^ Moreover, the availability of DXA is very limited, especially in developing countries.^5^ On the other hand, although FRAX is a widely available web‐based clinical risk assessment tool, its accuracy is limited by the exclusion of variables that are known to associate with fracture risks.^6^ Bone turnover markers (BTMs) such as serum procollagen N‐terminal propeptide (P1NP) and serum C‐terminal telopeptide of type I collagen (CTX) have emerged as useful tools for predicting and monitoring disease progression.^7^ In a recent meta‐regression analysis involving 28,000 participants enrolled in 14 trials, changes in BTMs were significantly associated with risk of vertebral fracture.^8^ However, the association was only significant in vertebral fracture but not in other fractures. Moreover, most of the BTMs are subject to diurnal variation.^9^ Therefore, research efforts have been diverted to identifying novel blood‐base bone biomarkers.

In the past decade, microRNAs (miRNAs) have been recognized as important regulators of bone metabolism. Compared with conventional BTMs, miRNAs often have a higher measurability and a more robust stability in body fluid.^10^ Therefore, they have a higher potential to be used as biomarkers for predicting and monitoring osteoporosis.^11^ Furthermore, miRNA‐based therapy, such as Miravirsen and MRX34, have already entered clinical trials for treating liver diseases and cancer, respectively. In bone research, improved in vivo osteogenesis was observed after miRNA treatment, highlighting the potential use of miRNAs as a therapeutic agent.^12^

Research in miRNAs and bone biology has grown rapidly over the past years, and studies on the roles of miRNAs in basic cellular functions were reviewed recently.^13^, ^14^ Eighty unique miRNAs were identified to associate with BMD, fracture, and osteoporosis in human studies (Supplemental Table S1). The current review will cover the advances in miRNA research on human osteoporosis from the following perspectives: 1) the effects of genetic variants and mutations on miRNA regulation in human; 2) the associations of miRNAs with osteoporosis and fractures; 3) the changes in miRNAs in response to osteoporosis medications.

## MicroRNA Biogenesis

MiRNAs are small non‐coding single‐stranded RNAs (19–24 bp). They regulate gene expressions by blocking translations and promoting degradations of target transcripts. There are at least 1400 mammalian miRNAs and 45,000 miRNAs target sites in human genome, covering 60% of the genes.^14^ Unlike the protein coding transcripts, these miRNAs are often coded in the intronic, intergenic, and polycistronic clustered regions.^15^

During miRNA biogenesis, primary miRNAs (pri‐miRNAs) can form multiple stem‐loop structures in the nucleus after transcription. In nucleus, the pri‐mRNA transcripts are trimmed into pre‐miRNAs by Drosha‐DGCR8 RNAse complex and are exported to cytoplasm through exportin‐5/GTP61. In cytoplasm, Dicer AGO2 and TRBP form a Dicer complex that further cleaves the pre‐miRNAs into miRNA duplexes. During miRNA guided gene suppression, one strand (guiding strand) of the miRNA duplex is loaded into a miRNA‐inducing silencing complex, which then binds to the 3′ untranslated region (3′ UTR) of target mRNA through Watson‐Crick pairing. The target mRNA will then either be subjected to translation inhibition or endonucleolytic cleavage.^16^ Each miRNA can regulate multiple target mRNA transcripts and each mRNA transcript can be regulated by different miRNAs, thus creating a complex regulatory network in numerous biological processes such as osteogenesis.

MiRNAs were shown to promote osteogenic differentiation of mesenchymal stem cells (MSCs) by suppressing osteogenic inhibitors or by mediating major osteoblastic differentiation and signaling pathways.^17^ Human studies on miRNAs over the past 8 years have established links between miRNAs and various skeletal phenotypes including BMD and fracture. Furthermore, studies on miRNAs from blood and bone tissue have provided important insights into fundamental cellular processes such as the differentiation of bone cells (osteoblast and osteoclast).^18^

## Effects of miRNA Polymorphisms on Skeletal Traits

Successful pairing of miRNAs’ 5′ seed region with target mRNAs’ 3′ UTR targeting site depends on two factors: sequence complementary of the two and secondary structures of target mRNAs. Hence, SNPs within or proximal to the loci of miRNA (miR‐SNPs) or mRNA (PolymiRTS) could potentially disrupt miRNA's targeting on mRNA. The potential effects of miR‐SNPs on skeletal traits have previously been discussed.^19^ Genetic variants in 7 miRNA families were reported to potentially affect skeletal traits, including miR‐27a, miR‐124, miR‐125, miR‐146a, miR‐149, miR‐196a2, and miR‐2861. Although many SNPs in these loci were originally reported in cancer research, subsequent human studies have provided further evidence to support their potential roles in bone biology.

Variants in miR‐146a, miR‐149, and miR‐196a2 were reported to associate with skeletal traits in multiple cohorts.^20^, ^21^, ^22^ Recent studies have further revealed several novel polymorphisms (let‐7g, miR‐140‐5p, miR‐149, miR‐3679, miR‐4274, miR‐433, miR‐499, and pri‐miR‐34b/c) that were significantly associated with skeletal traits.^22^, ^23^, ^24^, ^25^, ^26^, ^27^ To date, 13 miR‐SNPs were found to be associated with skeletal traits and they are summarized in Table 1.

**Table 1:** Genetic Variants Affecting miRNA Functions in Human

	Gene	miRNA	SNP/mutation	Allele	Description	Subject	Ref. no.
3′ UTR poly‐miRTSs	FGF2	miR‐146a/b	rs6854081	G/T	Significant association with femoral neck BMD (*p* = 8.37 × 10^−3^)	White (discovery *n* = 997; replication *n* = 1728)	20
		miR‐196a‐3p	rs1048201	C>T	Significant association with spine BMD (*p* = 1.53 × 10^−3^)	Chinese (discovery *n* = 1300; replication *n* = 1039)	21
	FGFRL1	miR‐140‐5p	rs4647940	C>G	Significant association with femoral neck BMD (*p* = 8.87 × 10^−12^)	Meta‐analysis of 7 cohorts	24
	ON	miR‐433	rs1054204	G>C	Allele G (Haplotype B) is associated with higher bone mass (*p* < 0.02)	White male (OP = 56 control = 59)	26, 41
	COL1A2	let‐7g	rs3917	INS/DEL	BMD was significantly increased in the INS/DEL or DEL/DEL group femoral neck (*p* = 0.018), total left hip (*p* = 0.028), L_1_ to L_4_ (0.025), and intertrochanteric area (*p* = 0.018)	Chinese (*n* = 487)	23
Exonic mutation	WNT1	miR‐18a‐3p	p.C218G (exon4)		Upregulated in mutation‐positive subjects	2 Finnish families with osteoporosis due to WNT1 p.C218G mutation (12 mutation‐postive; 12 mutation‐negative)	49
		miR‐223‐3p			Downregulated in mutation‐positive subjects		
		miR‐22‐3p					
		miR‐31‐5p					
		miR‐34a‐5p					
		miR‐143‐5p					
		miR‐423‐5p					
		miR‐423‐3p					
miR‐SNPs		pri‐miR‐34b/c	rs4938723	T>C	CC and CT/CC associated with a significantly reduced risk of OP (CC versus TT: OR = 0.32; *p* < 0.001; CT/CC versus TT: OR = 0.69; *p* = 0.016)	Chinese (*n* = 681)	27
		miR‐146a	rs2910164	C>G	miR‐146a CG/ miR‐196a2 TC combined genotype was more frequent in OVCF patients (OR = 5.163; *p* = 0.043)	Postmenopausal Korean women (*n* = 286)	22
		miR‐196a2	rs11614913	T>C			
		miR‐149a	rs2292832	T>C	TT genotype of miR‐149a T>C may contribute to decreased susceptibility to OVCF (OR = 0.435; *p* = 0.014)		
		miR‐499	rs3746444	A>G	miR‐146aG/‐149T/‐196a2C/‐449G allele combination may contribute to increased susceptibility of OVCF (OR = 35.01; *p* = 0.001)		
		miR‐3679	rs6430498	G>A	Significant association with low BMD (*p* = 0.001)	OSTEOMED2 cohort (*n* = 2183)	25
		miR‐4274	rs12512664	G>A			

^3' UTR polymiRTSs: Polymorphism in 3' untranslated region of microRNAs and their TargetSites; miR‐SNPs: Single nucleotide polymorphisms of microRNAs; BMD: Bone mineral density; OP: Osteoporosis; OVCF: Osteoporotic vertebral compression fracture; OR: Odds ratio; INS/DEL: Insertion/Deletion.^

## PolymiRTSs in FGF Signaling

### FGF2

Basic fibroblast growth factor (FGF2) is an essential mitogenic growth factor in the FGF polypeptide family. It is expressed in the majority of mesenchymal and bone‐related cells, including chondrocytes, osteoblasts, adipocytes, and osteolclasts. FGF2 is expressed in limb buds during the developmental stage and controls the growth and patterning of limbs. In bone cells, by regulating parathyroid hormone (PTH) and bone morphogenetic protein 2 (BMP2)‐induced bone formation,^28^, ^29^ FGF2 promotes bone marrow stromal cell differentiation into osteoblast.^30^ In osteoclast, FGF2 is required for the regulation of receptor activator of NF‐κB ligand (RANKL) and mesenchymal stem cell factor (M‐SCF)‐mediated differentiation.^31^ Therefore, FGF2 is a crucial regulator during both bone development and remodeling.

Two single‐nucleotide polymorphisms (SNPs; rs6854081 and rs1048201) at 3′ UTR of FGF2 were found to be associated with bone mineral density (BMD).^20^, ^21^ The minor allele G of rs6854081 was associated with low BMD in a cohort of white women (*n* = 2725).^20^ The study demonstrated that FGF2 transcript containing allele rs6854081‐T had a lower expression level in monocytes and B cells, and the allele was associated with higher BMD. The group further hypothesized that allele‐T at rs6854081 would result in a higher binding affinity with miR‐146a/b, hence suppressing FGF2 translation. Suppression of FGF2 would reduce osteoclastogenesis, which in turn leads to an increase in BMD. However, this causal relationship has not been experimentally validated yet. In another study, the minor allele T at rs1048201 was significantly associated with spine BMD in Han Chinese (*n* = 2339).^21^ Subsequent bioinformatics analysis and experimental validation suggested that miR‐196‐3p could affect both FGF2 mRNA and protein levels. Using dual‐luciferase assay and further Western blot confirmation in hFOB1.19 culture, they demonstrated rs1048201‐C had a repressive effect on FGF2 expression. However, the Han Chinese study failed to replicate the association between rs6854081 and BMD, suggesting the effect of rs6854081 and rs1048201 on bone regulation might be ethnic specific.

### FGFRL1

Given that FGF signaling has a ubiquitous role not only in skeletogenesis but also in the development and homeostasis of various cells, FGF pathway is tightly regulated. Fibroblast growth factor receptor like 1 (FGFRL1) is a non‐tyrosine kinase that provides additional control over FGF ligands’ activation. FGFRL1 functions by binding to several FGFs, including FGF2/3/8.^32^ Unlike the classical FGFRs, FGFRL1 is unable to cause downstream signal activation via tyrosine transphosphorylation.^33^ The mechanism of FGFRL1 regulation is unclear, though it is thought to function as a decoy receptor for FGFs or as a modulator of intracellular signaling transducers.^33^

Studies have shown that FGFRL1 could both promote and inhibit cell differentiation,^33^, ^34^, ^35^ although only a few studies have looked into the role of FGFRL1 in bone biology. A study on the differentiation of MSCs showed that FGFRL1 was a modulator of FGFR1/2.^32^ Interestingly, a more recent study demonstrated that during osteoblast differentiation, FGFRL1 acted as a positive regulator for FGFR2, but on the flip side, it could also promote adipocyte differentiation with FGFR1.^32^ This dual‐role nature of FGFRL1 supported further the idea of FGFR1 being a modulator during cell lineage commitment, although in‐depth research is still needed to uncover the mechanisms behind these regulations.

In a large‐scale meta‐analysis of 7 cohorts including white, Han Chinese, African Americans, and Hispanics, rs4647940 at FGFRL1's 3′ UTR was found to be significantly associated with BMD at the femoral neck.^24^ In this study, bioinformatics analyses showed that rs4647940 is located at an evolutionary conserved binding site of miR‐140‐5p. The binding affinity between miR‐140‐5p and FGFLR1 3′ UTR was further validated by dual luciferase assay. To test for the in vivo function of miR‐145‐5p, microinjection of dre‐miR‐140‐5p into zebrafish embryo was performed, and it resulted in an absence of ceratobranchial cartilages and severe craniofacial malformation. This suggested FGFRL1 and miR‐140‐5p could play an important role in bone formation and development.

## PolymiRTSs in ECM Formation

Bone extracellular matrix (ECM) proteins provide tensile strength and elasticity for bone tissue by serving as an essential framework for mineralization. Collagen type I contributes to 90% of the ECM proteins, whereas collagen type I alpha 2 (COL1A2) is a major component of collagen type I triple helix fiber.^36^ The assembly of collagen fibers are aided by non‐collagenous matrix proteins, such as osteonectin (ON).^37^ ON is one of the most abundant non‐collagenous matrix proteins. Besides matrix organization, ON promotes osteoblast differentiation and survival in MSCs while supressing adipogenesis.^38^, ^39^ Therefore, COL1A2 and ON are essential for proper bone formation and mineralization, and mutation in either of the genes could result in major skeletal disorders such as osteogenesis imperfecta.^40^

Human studies on miRNAs and polymiRTSs of COL1A2 and ON has provided insight into the miRNA species that affect bone extracellular matrix (ECM). A study (*N* = 487) reported that an insertion/deletion (INS/DEL) polymorphism (rs3917) in the 3′ UTR of COL1A2 was associated with risk of osteoporosis.^23^ The study demonstrated a significant reduction in BMD in the INS/DEL or DEL/DEL group versus the INS/INS group; subsequent in vitro study using 48 patients’ primary osteoblast showed that let‐7g could negatively regulate COL1A2 in osteoblast with INS/INS genotype but not with INS/DEL and DEL/DEL genotypes. Notably, the findings were contradictory: INS/INS genotype was associated with a reduced expression of COL1A2 in primary osteoblast, yet it was also associated with higher BMD in human. Additionally, the mineralization ability of the primary osteoblasts was not evaluated. Thus, further study is required to examine the mechanistic relation of rs3917 to bone metabolism.

For ON, rs1054204, which is located in the 3′ UTR of ON, was associated with BMD in middle‐aged white men with idiopathic, low turnover osteoporosis (*n *= 56).^41^ Two haplotypes at the ON 3′ UTR were identified: haplotype A with a G allele at rs1054204 and haplotype B with a C allele at rs1054204. It was observed that rs1054204‐C was associated with higher BMD. The finding was validated independently using in vivo mice model. Mice with rs1054204‐C had increased cortical bone area and *ON* expression. MiR‐433 was suggested to be a potential regulator of ON. The expression of miR‐433 was shown to be reducing during osteoblast differentiation, and direct interaction between miR‐433 and rs1054204 was observed via ON 3′ UTR reporter constructs.^26^

## MiRNAs in WNT1 Mutation

Canonical WNT/β‐catenin signaling is a well‐established pathway regulating bone formation and remodeling. In vitro studies have identified multiple miRNA binding partners of key Wnt signaling components, such as LRP‐6 (miR30e‐5p), DKK1 (miR‐152‐3p, miR‐335), and APC (miR‐27a‐3p, miR‐142).^42^, ^43^, ^44^, ^45^, ^46^, ^47^ Although the effects of Wnt signaling components in bone have been widely studied, the relationships between various Wnt ligands and miRNAs in bone biology is still unexplored. WNT1 is one of the Wnt ligands. In human, monoallelic mutation at WNT1 would lead to inherited early onset of osteoporosis, whereas biallelic mutations would lead to osteogenesis imperfecta.^48^

So far, the role of miRNA in monogenetic bone diseases remained largely unexplored. A recent study investigated the relationship between serum miRNA profiles and BMD in individuals with heterozygous WNT1 p.C218G mutation.^49^ The study was conducted in 24 subjects from two Finnish families, in which half of the subjects were mutation positive. In the mutation‐positive subjects, p.C218G missense mutation resulted in early onset and progressive osteoporosis with normal levels of BTMs. The study showed that 6 miRNAs were significantly downregulated (miR‐22‐3p, miR‐31‐5p, miR‐34a‐5p, miR‐143‐5p, miR‐423‐3p, and miR‐423‐5p) in the osteoporotic subjects. MiR‐31‐5p had no known function in WNT1, but it was reported to be involved in osteogenesis;^50^, ^51^ miR‐423‐3p/5p were not linked to bone metabolism or WNT signaling. For the remaining miRNAs, miR‐22‐3p, miR‐34a‐5p, and miR‐143‐5p were known to target WNT signaling molecules or bone‐related genes such as RUNX2, Osterix, and WNT1.^52^, ^53^, ^54^ On the other hand, miR‐18a‐3p and miR‐223‐3p were found to be significantly upregulated. Among them, miR‐223‐3p was known to target bone‐related genes.^55^, ^56^ This Finnish study was the first to evaluate miRNA profiles in WNT1 heterozygous mutation subjects. However, whether these differentially expressed miRNAs could serve as bone biomarkers is still unclear and further investigations are warranted.

## MiR‐SNPs

Genetic variants affecting miRNAs’ functions were not found only in target mRNA transcripts. The number of reports on miR‐SNPs, ie, SNPs located within the pri/pre‐miRNA and promoter sequence, have been increasing over the past years. MiR‐SNPs could alter miRNA maturation and its target binding affinity. Certain variants could also trigger alternative cleavage for miRNAs’ biogenesis enzymes, leading to abnormal miRNA expression or even new miRNA isoforms.^57^

### MiR‐34b/c and TP53

MiR‐34b and miR‐34c belongs to the miR‐34 family, which consists of miR‐34a, miR‐34b, and miR‐34c. MiR‐34b and miR‐34c shares the same primary transcript at chromosome 11q23, whereas miR‐34a is located at chromosome 1p36. Members of the miR‐34 family are downstream targets of TP53, a well‐known cancer suppressor.^58^ The miR‐34 family are involved in a wide range of cellular process.^58^ Targets of miR‐34 include components of major bone signaling pathways such as Notch and Wnt signaling.^59^, ^60^, ^61^, ^62^ In vivo mice models showed that miR‐34c regulated osteoclast differentiation by targeting multiple Notch components including Notch1/2 and Jag. Additionally, miR‐34b was reported to play a causal role in osteosarcoma.^63^ MiR‐34a was associated with WNT1 mutation in the human study aforementioned^49^ and miR‐34a KO mice exhibited elevated bone resorption and reduced bone mass.^64^

Rs4938723 is located at the CpG region of pri‐miR‐34b/c promoter. This variant of miR‐34b/c was known to associate with cancers.^65^, ^66^ In a recently study, rs4938723 miR‐34b/c was also found to be associated with osteoporotic risk.^27^ The study investigated the association of mutations at rs4938723 and TP53 (Arg72Pro) with osteoporosis among 310 osteoporosis patients and 371 controls. A significant reduction of osteoporosis risk was observed for homozygous CC at rs4938723 (odds ratio [OR] = 0.35, 95% confidence interval [CI] 0.19–0.64; *p* < 0.001), whereas the TP53 Arg72Pro CC genotype carriers showed a significant increased risk of osteoporosis (OR = 2.21, 95% CI  1.45–3.37; *p* < 0.001).

### MiR‐146a, 149, 196a, and 499

Rs2910164 (miR‐146a), rs2292832 (miR‐149), rs11614913 (miR‐196a‐2), and rs3746444 (miR‐499) are known to associate with various diseases.^67^, ^68^, ^69^ In bone, miR‐196a and miR‐146a were involved in osteoblast and osteoclast differentiation,^70^, ^71^ whereas miR‐146a and miR‐149 were linked to rheumatoid arthritis (RA) and osteoarthritis (OA), respectively.^72^, ^73^ The association of these SNPs with osteoporotic fracture was evaluated in a recent study composed of 286 postmenopausal Korean women (57 with osteoporotic vertebral compression fracture [OVCFs], 55 with non‐OVCFs, and 174 healthy controls).^22^ It was reported that a combination of genotypes miR‐146aG/‐149T/‐196a2C/‐449G was associated with increased OVCF risk (OR = 35.01; 95% CI 1.919–638.6, *p* = 0.001). In contrast, miR‐149a T/T was associated with a reduced risk (OR = 0.435; 95% CI 0.22–0.85, *p* = 0.014). Although these SNP‐containing miRNAs were seldom reported in other human studies of bone fractures, investigations using cell or animal models have supported their significance in bone metabolism.

### MiR‐146

The miR‐146 family consists of miR‐146‐a and −b and they are both linked to polymiRTSs of FGF2 as discussed previously. MiR‐146a, located at 5q33.3, is expressed in cartilage and femur and other non‐skeletal tissues including liver, breast, and hematopoietic cells. During bone formation, miR‐146a plays a positive role by targeting SMAD4 in the TGF‐β signaling, which in turn suppresses SOX9 and increases RUNX2 expression.^74^ Increased miR‐146a expression contributes to osteoarthritis, which was postulated to be caused by reduced chondrocyte survival via TGF‐β suppression.^75^ On the other hand, miR‐146b, located at 10q24, drives MSCs toward adipogenesis.^76^ In human skeletal stem cells, miR‐146b is downregulated during chondrogenic differentiation. High level of miR‐146b contributes to osteoarthritis by suppressing SOX5, which is an important transcription factor in chondrogenesis.^77^

### MiR‐196

The miR‐196 family is located within the HOX clusters. HOXs are major regulators of skeletal patterning and limb development and they coordinate morphogen signaling pathways such as BMP and TGF.^78^ MiR‐196 regulates skeletal development by orchestrating the level of HOX proteins in a temporal spatial manner.^79^, ^80^

For miR‐196a, it was shown to drive MSCs toward osteoblast lineage by targeting HOXC8.^81^ The 3p strand, miR‐196a‐3p, was also shown to interfere with FGF2 expression in a human study on FGF2 polymiRTS.^21^ Within the miR‐196a‐2 sequence, like many other miRNAs, rs11614913 was first reported to be associated with cancer susceptibility.^82^, ^83^ Recent in vivo studies using mouse model revealed a co‐regulatory mechanism of the miR‐196 family and HOX genes in controlling vertebral morphology.^84^ Using data from the GEFOS consortium and the Rotterdam study, significant associations between rs11614913 and both lumbar spine and femoral neck BMD were reported in female subjects.^85^

### MiR‐149 and 499

MiR‐149 and miR‐499 have not been shown to be directly involved in bone metabolism. Previous human and in vitro studies reported their roles in inflammatory response: MiR‐149 and 499 are both involved in the suppression of inflammatory cytokines including IL‐1β, IL‐6, and TNF‐α.^72^, ^86^ The SNP rs3746444 in miRNA‐499 was shown to associate with rheumatoid arthritis in a meta‐analysis.^87^ MiR‐149 was downregulated in an in vitro osteoarthritis model upon inflammatory stimulation.^72^ The SNP rs2292832 in miR‐149 was shown to associate with breast cancer and ischemic stroke;^69^, ^88^ however, its association with BMD in human has not been reported in literature so far.

### MiR‐3679 and 4274

MiRNA species in skeletal research often lack functional characterization. MiR‐3679 and miR‐4272 are novel miRNAs identified from a cohort of Spanish postmenopausal women.^25^ The allele A of rs6430498 in pre‐miR‐3679 and rs12512664 in pre‐miR‐4234 were associated with decreased BMD at femoral neck in the OSTOMED2 cohort (*n* = 2183). The study also observed overexpression of both miR‐3679 and miR‐4234 in trabecular bone samples from osteoporotic fracture patients. In functional study, miR‐3679 inhibition was shown to increase matrix mineralization by 10% in primary hOB cells.^(25)^ Given that studies on miR‐3679 and miR‐4274 in bone metabolism are scarce, further validation for the mechanistic roles of miR‐3679 and miR‐4274 as well as their effects is needed.

## MiRNAs in Human Osteoporosis Studies

Human osteoporosis studies on miRNAs thus far have identified 57 miRNA families that were potentially related to bone metabolism (Supplemental Table S1). Studies on the same pathological conditions often detected partially overlapping or, in some cases, completely new miRNA signatures,^89^, ^90^ thus increasing the number of novel miRNAs that could potentially affect bone metabolism. However, many still need further characterization. In the following section, miRNAs that were identified across multiple human studies will be reviewed (Table 2).

**Table 2:** Overview of Major miRNAs in Human Studies

miRNA	Level changes in primary osteoporotic subjects		Population	Sample size	Phenotypes and targets in related functional studies	Ref. no.
miR‐21	Increase	Serum and bone tissue	German	*n* = 30 per group (serum); *n* = 20 per group (bone tissue)	PDCD4 (osteoclast, mouse); PI3K/β‐catenin pathway (human umbilical cord MSCs)	89
		Serum; bone tissue; isolated osteoblast osteoclast	German	*n* = 7 per group		91
	Decrease	Plasma	Chinese postmenopausal women	*n* = 40 per group (osteoporosis versus osteopenia versus normal)	Stimulate osteogenesis (hPDLSCs)	92
		Serum level is associated with increased vertebral fracture and lower bone mass	Greek postmenopausal women	*n* = 70 versus 30 control		93
miR‐23‐3p	Increase	Serum and bone tissue	German	*n* = 30 per group (serum); *n* = 20 per group (bone tissue)	Reduce bone mass (mouse—gain of function)	89
	Decrease	Serum level is associated with increased vertebral fracture and lower bone mass	Greek postmenopausal women	*n* = 70 versus 30 control	Stimulate osteogenesis (hBMSCs)	93
miR‐100	Increase	Serum and bone tissue	German	*n* = 30 per group (serum); *n* = 20 per group (bone tissue)	BMPR2, SMAD1 (mouse MSC)	89
	Decrease	Serum; bone tissue; isolated osteoblast osteoclast	German	*n* = 7 per group		91
miR‐124	Increase	Serum and bone tissue	German	*n* = 30 per group (serum); *n* = 20 per group (bone tissue)	Dlx2,3,5 (mouse MSC)	89
	Decrease	Serum level is associated with increased vertebral fracture and lower bone mass	Greek postmenopausal women	*n* = 70 versus 30 control	NFATc1, Rab34 (mouse bone marrow monocytes)	93
miR‐125b	Increase	Serum and bone tissue	German	*n* = 30 per group (serum); *n* = 20 per group (bone tissue)	Cbfβ (C3H10T1/2 cells)	89
		Serum; bone tissue; isolated osteoblast osteoclast	German	*n* = 7 per group		91
		Serum	Spanish	*n* = 12 versus 15 osteoarthritic control		108
		Expression in bone biopsy	Postmenopausal women (Array Express database)	*n* = 27 versus 39 control		109
miR‐133	Increase	Serum	White (US) postmenopausal women	*n* = 10 per group	Osteoclastogenesis (R264.7, THP‐1 cells) Increased bone mass (knockout mouse)	113
		Plasma	Chinese postmenopausal women	*n* = 40 per group (osteoporosis versus osteopenia versus normal)		92
		Serum	Chinese postmenopausal women	*n* = 10 per group		114

### MiR‐21

MiR‐21 is one of the most frequently identified miRNA species in human bone researches. Over the past years, independent studies have suggested that changes in circulating miR‐21 levels are significantly associated with osteoporotic risk. An early study found that miR‐21 levels were elevated in serum (*n* = 30 per group) and bone tissue (*n* = 20 per group) of osteoporotic subjects.^89^ Similarly, another study showed a significant increase in circulating miR‐21‐5p in osteoporotic subjects (*n* = 7 per group).^91^ Its expression was also upregulated in isolated bone tissues, primary cultures of osteoblast and osteoclast. These results suggested that miR‐21 was positively correlated with risk of osteoporosis. However, other studies reported opposite findings. In a study of Chinese postmenopausal women with osteopenia and osteoporosis, plasma miR‐21 level was significantly reduced (*n *= 40 per group).^92^ A later study on the osteoporotic individuals in the Greek population also reported that decrease in serum miR‐21‐5p was significantly associated with increased risk of vertebral fracture and low bone mass.^93^ These contradictory results in human studies may be attributable to relatively small sample sizes.

In vitro studies showed that miR‐21 had a role in both osteoblast and osteoblast biology. In osteoclast, miR‐21 was shown to promote osteoclastogenesis by downregulating PDCD4, which would in turn de‐represses c‐Fos, a key transcription factor for macrophage‐osteoclast differentiation.^94^ Similarly, a recent study using miR‐21 KO mouse model also supported the role of miR‐21 as an osteoclastogenic promoter.^95^ MiR‐21^‐/‐^ mice showed protection against age‐related osteopenia with increased PDCD4 level and trabecular bone mass accrual despite the elevated serum RANKL. However, in osteoblast differentiation, the role of miR‐21 remained unclear. MiR‐21 was reported to stimulate osteoblastic differentiation of MSCs through the PI3K/β‐catenin pathway in human umbilical cord MSCs.^96^ Yet, in human periodontal ligament stem cells (hPDLSCs), decreased expression of miR‐21 was observed during osteogenesis.^97^ At this point, evidence from functional studies showed consistently a positive role of miR‐21 in osteoclastogenesis; however, its role in osteoblast differentiation remained controversial.

### MiR‐23‐3p

For mir‐23‐3p, thus far, no clear conclusion on the role of miR‐23 could be drawn from human studies. Seeliger and colleagues showed an increased miR‐23a‐3p level in both serum and bone tissue of osteoporotic patients.^89^ However, a recent study of osteoporotic postmenopausal women (*n* = 70) versus control (*n* = 30) yielded conflicting results, in which circulating miR‐23a‐3p was significantly lower in individuals with low bone mass.^93^

In vitro study on human bone marrow mesenchymal stem cells (hBMSCs) revealed that increased miR‐23a/b expression could promote osteoblastic differentiation.^98^ Moreover, it was further shown in vivo that continuous stimulation on differentiation could eventually lead to bone mass reduction, where increased level of miR‐23a in osteoblast could promote its terminal differentiation to osteocyte.^99^ Mice with gain‐of‐function mutation of miR‐23a cluster (Col1a1‐miR‐23aC) appeared to have lower bone mass. Such phenotype could be explained by a reduced osteoblast number and mineralized surface as well as an increased number of osteocytes.

### MiR‐100

After the identification of miR‐100 in an early human study of osteoporotic serum and bone tissue samples,^89^ a subsequent study conducted by the same group demonstrated further that osteoporotic patients had a significant upregulation of miR‐100‐5p in both isolated osteoblast and osteoclast.^91^ In ex vivo cultures of patients’ osteoblast, higher miR‐100‐5p expression was observed during a 14‐day culture period. In osteoclast, a significant increase in miR‐100‐5p expression was observed after 28 days of culture. Furthermore, the study demonstrated a strong inverse correlation between miR‐100‐5p expression in bone tissue and BMD at the femoral neck. These results suggested that an overall increase in miR‐100‐5p level might lead to bone loss.

An independent in vitro study of MSC revealed that miR‐100 expression was reduced during osteoblast differentiation.^(100)^ Overexpression of miR‐100 in MSC was shown to inhibit osteogenic differentiation by acting as an attenuator for key signaling molecules in osteogenic differentiation, including morphogenetic protein receptor type II (BMPR2) and SMAD1.^100^, ^101^ On the other hand, the role of miR‐100 in osteoclastogenesis has yet to be studied, and the interaction between different bone cells in in vivo environment under the effect of miR‐100 is still largely unknown. So far, the evidence has been consistent that miR‐100 could inhibit bone formation.

### MiR‐124

Human studies shown that serum miR‐124 levels were elevated in patients with low bone mass.^89^, ^93^ MiR‐124 could negatively regulate osteoblast formation by targeting essential osteogenic inducers, including Dlx2, Dlx3, and Dlx5. Moreover, expression of miR‐124 decreased during osteoblast differentiation and overexpressing miR‐124 mimic drove MSC to adipogenic differentiation.^102^ MiR‐124 also targeted essential transcription factor for osteoclastogenesis (NFATc1)^103^ and major regulator of secretory lysosomes (Rab27a),^104^, ^105^ which resulted in suppressing monocyte proliferation and differentiation.^105^ Besides bone cells, miR‐124 was also shown to inhibit myogenic differentiation in MSC, which would potentially lead to increased risk of fracture in elderly by escalating muscle loss.^106^ Overall, miR‐124 exhibited inhibitory effects on stem cell differentiation. Additional evidence from a recent study that an increase in miR‐124 expression was observed in aged skin cells^107^ suggests miR‐124 might play a role in diverse aging processes as well.

### MiR‐125b

Like miR‐100 and miR‐124, miR‐125b was suggested to exert inhibitory effect on osteogenesis. Multiple studies reported an increased circulating miR‐125b expression in osteoporotic patients.^89^, ^91^, ^108^, ^109^ MiR‐125b‐5p level was found to be inversely correlated with BMD at the femoral head, with an increased expression being observed in osteoblast and osteoclast from osteoporotic patients.^91^ When comparing the circulating miRNAs in patients with osteoporotic fracture with those in osteoarthritic controls, circulating miR‐125b‐5p was also significantly upregulated in the osteoporotic fracture group.^108^

To date, the functional role of miR‐125b in bone metabolism remained largely unknown. In C3H10T1/2 cells, overexpression of miR‐125b inhibited osteoblast differentiation by targeting Cbfβ, a key transcription osteogenic inducer, and in turn reduced Runx2 expression.^110^ However, this effect was not observed in another study using human MSCs.^111^ No study so far has evaluated miR‐125b's role in osteoclastogenesis.

### MiR‐133

Because of estrogen deficiency and increased calcium loss, postmenopausal women are more susceptible to osteoporosis and possess a higher fracture risk than men,^112^ and miRNAs may play a role in postmenopausal osteoporosis. Association between miR‐133a and BMD variation in osteoporotic postmenopausal women has been reported in multiple studies. By measuring the miRNA expression in circulating monocytes in white postmenopausal women, Wang and colleagues found a significant increase in miR‐133a expression in low BMD group.^113^ Using bioinformatics analysis, osteoblast‐related genes, such as CXCL11, CXCR3, and SLC39A1, were found to be potential downstream targets. Two subsequent studies in Chinese postmenopausal women also reported increased miR‐133a levels in serum and plasma of low BMD group.^92^, ^114^

Liz and colleagues provided evidence that miR‐133a could contribute to RANKL‐induced osteoclast differentiation.^114^ By overexpressing miR‐133a in R264.7 and THP‐1 cells, expressions of osteoclastogenesis markers (NFATc1, c‐Fos, and TRAP) were induced, whereas in miR‐133a knockdown, expressions of these markers were suppressed. Similarly, in ovariectomized rats, miR‐133a knockdown led to a significant increase in lumbar spine BMD. A study on arterial calcification provided clues for the effects of miR‐133a on osteoblast. Overexpression of miR‐133a inhibited transdifferention of vascular smooth muscle cells into osteoblast‐like cell by targeting RUNX2 directly.^115^ Taken together, these studies suggested that miR‐133a could promote bone resorption and potentially inhibit bone formation.

Other miRNAs such as miR‐194 and miR‐19a were also proposed to be biomarkers for postmenopausal osteoporosis.^116^, ^117^ However, there was no functional study or replication study conducted, thus their roles in bone metabolism remained inconclusive.

## MiRNAs in Secondary Osteoporosis

### Glucocorticoid

Glucocorticoid‐induced osteoporosis (GIO) is the most common and severe form of secondary osteoporosis. A recent study evaluated the mRNA and miRNA levels in bone samples from Cushing's disease patients with endogenous hypercortisolism.^118^ Elevated cortisol level is known to associate with accelerated bone loss in both men and women.^119^ In line with current understanding, among the hypercortisolism patients, bone marker genes, such as RUNX2, BGLAP, and ALPL, were downregulated, whereas expressions of Wnt‐signaling antagonists were found increased. Furthermore, miRNAs that could impair bone formation (miR‐34a‐5p, miR‐125b‐5p and miR‐188‐3p) were also found upregulated.

### Autoimmune disorders

Glucocorticoid therapy used in treating autoimmune diseases, such as inflammatory bowel disease (IBD) and systemic lupus erythematosus (SLE), could lead to osteoporosis.^120^, ^121^ IBD patients are known to have elevated proinflammatory circulating cytokine and increased risk of fracture.^122^ A study investigating the miRNA expressions in peripheral immune cells of ulcerative colitis patients with low BMD found that miR‐19a was significantly associated with inflammatory marker lL‐13.^123^ MiR‐19a was previously found to be upregulated in postmenopausal osteoporosis.^117^ However, whether miR‐19a played a role in inflammation‐mediated bone metabolism is still unclear and it requires further study.

SLE is a complex immune disorder, and osteoporosis is a well‐known comorbidity of SLE. MiR‐148a was found overexpressed in T cells of lupus patients.^124^ Because miR‐148a is known to directly suppress MAFB, a repressor for RANKL‐induced osteoclastogenesis, increased miR‐148a could de‐repress osteoclast differentiation and potentially contribute to bone loss. In fact, Peng and colleagues found an increase in miR‐148a expression in CD14+ peripheral blood mononuclear cells among lupus patients with low BMD.^125^ Additionally, CD14+ PBMC from these patients also possessed increased TRAP activity, highlighting the potential role of miR‐148a in enhancing osteoclastogenesis.

### Type 2 diabetes

Type 2 diabetes (T2D) patients often have higher BMD and increased fracture risk both at the same time.^126^ This could be because of poor bone quality, another important determinant of bone strength.^127^, ^128^ In a study on the relationship between diabetic bone fracture and miRNAs, Ursula and colleagues investigated the circulating miRNA profiles of 80 postmenopausal women with T2D and osteoporosis and found that miR‐550a‐5p, miR‐96‐5p, miR‐382‐3p, and miR‐181c‐5p were associated with fracture risk.^129^ The finding suggested that aging acceleration was involved and it could be caused by increasing cellular oxidative stress and dysregulating lipid and glucose metabolism in human body.^130^, ^131^ They further investigated the effects of miR‐550a‐5p and miR‐382‐3p on bone metabolism and found that while both miR‐382‐3p and miR‐550a‐5p impaired adipogenic differentiation, respectively they significantly enhanced and inhibited osteogenic differentiation in human adipose‐derived stem cells (hASCs).

To date, there are only a handful of human studies investigating the miRNA signatures of secondary osteoporosis. More studies on the secondary causes of osteoporosis, such as hyperthyroidism, chronic liver diseases, and hematological disorders, are needed.

## MiRNAs in Antiosteoporosis Treatment

Teriparatide (TPTD) and denosumab (Dmab) are currently two of the most potent anti‐osteoporotic medications.^132^, ^133^ Teriparatide is an anabolic agent that mimics human parathyroid hormone, which promotes calcium absorption and bone formation. Denosumab is an antiresorptive agent that specifically binds to RANKL, prevents RANK activation and osteoclast formation, and results in decreased bone resorption.

The changes in miRNA expressions during teriparatide and denosumab treatments were recently investigated.^134^, ^135^ Because the two drugs have opposite effects, distinct miRNA profiles were observed in serum from postmenopausal women who were undergoing either TPTD or Dmab treatment.

In the TPTD treatment group, miR‐133a and miR‐33 levels were significantly decreased after 3 to 12 months of treatment. As shown in other human studies, miR‐133a could promote osteoclast differentiation, and its level was correlated with low BMD in postmenopausal women.^92^, ^113^, ^114^ It was also shown that miR‐133a could hamper bone formation by targeting RUNX2.^136^ MiR‐33, on the other hand, was involved in mechano‐induced osteoblast differentiation,^137^ but its association with BMD has yet to be reported.

In the Dmab treatment group, no significant change in miRNA levels were observed during treatment.^134^ However, after denosumab treatment was ceased, a significant change in miRNA levels took place, and this change might contribute to the increased fracture risk observed among patients after stopping denosumab treatment.^135^ In individuals who suffered these rebound‐associated fractures, significantly lower circulating miR‐503 and miR‐222 were observed. These changes were associated with a drastic increase in the expressions of osteoclastogenesis‐related genes RANK (13‐fold) and CTSK (2.6‐fold), indicating a significant rebound in osteoclast activity after denosumab discontinuation. Functionally, miR‐503 was shown to suppress Smuf1 expression and promote bone formation.^138^ On the other hand, miRNA‐222 could be a negative regulator of osteogenesis because inhibition of miR‐222 was shown to enhance fracture healing.^139^ Although the underlying mechanisms of these changes remained unknown, these discoveries would serve as an important starting point for further investigations into the roles of miRNAs in anti‐osteoporotic treatment.

## Conclusion

Human studies on the relationship between miRNAs and bone metabolism have provided valuable insights into the roles of miRNAs in osteoporosis. Over the years, using bone tissues, serum, plasma, and circulating blood cells of individuals with different skeletal conditions, population‐based studies have been continuously updating the library for bone metabolism–related miRNAs. Although the clinical translation of miRNAs in anti‐osteoporotic treatment is still at its infancy and many of the miRNAs still require extensive validation and characterization, current research efforts have already kick‐started the development of miRNA‐based fracture risk assessment tools like osteomiR, which was shown to effectively reduce screening and monitoring costs for osteoporosis.^11^ Yet, because of limited sample sizes and unreliable bone measurements, identification of clinically useful miRNAs for osteoporosis management remains a challenging task. Large‐scale collaborative effort is warranted for a larger study sample and to provide a consensus on the methodology in human studies, in hopes of delineating the complex relationship between miRNAs and bone metabolism.

## Disclosures

All authors state that they have no conflict of interest regarding this manuscript.

## Supporting information

Supporting Table S1.
