# Supplementary material for: MicroRNA and Human Bone Health
Source: JBMR Plus. 2018 Nov 5;3(1):2–13. doi: 10.1002/jbm4.10115 (PMC6339549; doi:10.1002/jbm4.10115)
Supplement: Supplementary file 1 — Supporting Table S1. [file JBM4-3-2-s001.docx]

Supplementary table 1: List of miRNAs in human studies

| Family | miRNA | Human studies (PMID) |
| --- | --- | --- |
| let 7g | let 7g | 27665867 |
| let 7g | let-7g-5p | 26026730 |
| miR-100 | miR-100 | 24431276 |
| miR-100 | miR-100-5p | 29158518 |
| miR-122 | miR-122-5p | 26163235 |
| miR-122 | miR-122a | 24431276 |
| miR-124 | miR-124 | 27836951 |
| miR-124 | miR-124-3p | 29309589 |
| miR-124 | miR-124a | 24431276 |
| miR-125 | miR-125 | 26329309 |
| miR-125 | miR-125b | 24431276 |
| miR-125 | miR-125b-5p | 29158518 |
| miR-125 | miR-125b-5p | 26163235 |
| miR-1270 | miR-1270 | 29285077 |
| miR-133 | miR-133a | 29425279 |
| miR-133 | miR-133a | 29309589 |
| miR-133 | miR-133a | 25231354 |
| miR-133 | miR-133a | 22506038 |
| miR-140 | miR-140-5p | 27552543 |
| miR-140 | miR-140-5p | 25941324 |
| miR-143 | miR-143-5p | 29506076 |
| miR-144 | miR-144-3p | 29334613 |
| miR-146 | miR-146a | 28741852 |
| miR-146 | miR-146a | 20641033 |
| miR-146 | miR-146b | 20641033 |
| miR-148 | miR-148a | 24431276 |
| miR-148 | miR-148a | 23225151 |
| miR-148 | miR-148a-3p | 27900532 |
| miR-149 | miR-149 | 28741852 |
| miR-152 | miR-152-3p | 27552543 |
| miR-187 | miR-187 | 25432767 |
| miR-18 | miR-18a-3p | 29506076 |
| miR-194 | miR-194-5p | 29196685 |
| miR-194 | miR-194-5p | 26038726 |
| miR-196 | miR-196a2 | 28741852 |
| miR-196 | miR-196a-2 | 29186852 |
| miR-196 | miR-196a-3p | 28317323 |
| miR-19 | miR-19a | 27381199 |
| miR-19 | miR-19a-3p | 27552543 |
| miR-19 | miR-19b-3p | 27552543 |
| miR-21 | miR-21 | 27836951 |
| miR-21 | miR-21 | 25882990 |
| miR-21 | miR-21 | 25231354 |
| miR-21 | miR-21 | 24431276 |
| miR-214 | miR-214 | 23223004 |
| miR-21 | miR-21-5p | 29309589 |
| miR-21 | miR-21-5p | 29158518 |
| miR-21 | miR-21-5p | 27836951 |
| miR-21 | miR-21-5p | 26163235 |
| miR-221 | miR-221 | 28123639 |
| miR-222 | miR-222-2 | 28283537 |
| miR-222 | miR-222-5p | 29309589 |
| miR-223 | miR-223-3p | 29506076 |
| miR-22 | miR-22-3p | 29506076 |
| miR-22 | miR-22-3p | 26026730 |
| miR-23 | miR-23 | 27836951 |
| miR-23 | miR-23a | 24431276 |
| miR-23 | miR-23a-3p | 29309589 |
| miR-24 | miR-24 | 26329309 |
| miR-24 | miR-24 | 24431276 |
| miR-24 | miR-24-3p | 29309589 |
| miR-25 | miR-25 | 24431276 |
| miR-25 | miR-25-3p | 27821865 |
| miR-26 | miR-26a-5p | 29309589 |
| miR-27 | miR-27a | 29309589 |
| miR-27 | miR-27a | 27337099 |
| miR-2861 | miR-2861 | 27836951 |
| miR-29 | miR-29 | 27836951 |
| miR-30 | miR-30e-5p | 27552543 |
| miR-31 | miR-31-5p | 29506076 |
| miR-320 | miR-320a | 28535813 |
| miR-324 | miR-324-3p | 27552543 |
| miR-328 | miR-328-3p | 26026730 |
| miR-331 | miR-331 | 26329309 |
| miR-33 | miR-33-3p | 29309589 |
| miR-335 | miR-335-5p | 29309589 |
| miR-335 | miR-335-5p | 27552543 |
| miR-34 | miR-34a-5p | 29506076 |
| miR-355 | miR-355 | 24582835 |
| miR-3679 | miR-3679 | 28364128 |
| miR-382 | miR-382-3p | 27345526 |
| MiR-422 | MiR-422a | 24820117 |
| miR-423 | miR-423-3p | 29506076 |
| miR-423 | miR-423-5p | 29506076 |
| miR-4274 | miR-4274 | 28364128 |
| miR-433 | miR-433 | 25262637 |
| miR-483 | miR-483-5p | 28535813 |
| miR-499 | miR-499 | 28741852 |
| miR-503 | miR-503 | 28283537 |
| miR-503 | miR-503 | 23821519 |
| miR-518 | miR-518f | 25432767 |
| miR-548 | miR-548×-3p | 29285077 |
| miR-550 | miR-550a-3p | 27552543 |
| miR-550 | miR-550a-5p | 27345526 |
| miR-8084 | miR-8084 | 29285077 |
| miR-93 | miR-93 | 24431276 |
| miR-93 | miR-93-5p | 29158518 |
| miR-34 | pri-miR-34b/c | 27227383 |
